# Being screened for frailty in the emergency department: the voice of patients in an exploratory qualitative study

**DOI:** 10.1186/s12877-026-06990-1

**Published:** 2026-01-16

**Authors:** Erika Hörlin, David Ekermo, Daniel Wilhelms, Ann Catrine Eldh

**Affiliations:** 1 Clinical Department of Emergency Medicine in Linköping, Region Östergötland, Linköping, Sweden and Department of Biomedical and Clinical Sciences, Linköping University, Linköping, Sweden; 2 Department of Health, Medicine and Caring Sciences, Linköping University, Linköping, Sweden

**Keywords:** Older adults, Patient experience, Emergency medicine, Clinical frailty scale

## Abstract

**Background:**

Frailty assessment is recommended in the emergency department (ED). Despite the widespread use of the Clinical Frailty Scale (CFS), little is known about how patients experience being assessed for frailty in an ED setting. The aim was to investigate patients’ experiences of frailty assessment with the CFS at the point of ED care.

**Methods:**

This study adopted a qualitative design, including video-recorded clinical encounters in which the CFS assessment was performed, as well as semi-structured interviews with patients. For analysis, thematic analysis was conducted.

**Findings:**

A total of 21 patients aged ≥65 years were recruited using purposive sampling. Four themes were identified—namely, Shaping the CFS experience, Meaning of the assessment, Missing the point, and Reflecting on ageing. In general, older patients viewed CFS assessment in the ED positively or indifferently. They highlighted the importance of the staff employing active listening and building rapport during the assessment, as this made the patients feel comfortable and willing to share information about their situations. Although patients occasionally struggled to understand the purpose and consequences of the assessment, they interpreted its meaning as relating to their health status or need for assistance.

**Conclusion:**

Most older ED patients experience CFS assessment as positive or indifferent, underscoring the importance of relationship-oriented communication to build trust and ensure quality in the frailty screening. The findings further emphasise the need for clarification of the purpose and potential outcomes of the assessment, as these were not always clear to patients.

**Registration:**

The study was registered at ClinicalTrials.gov (identifier: NCT06621290) on September 30, 2024.

**Supplementary Information:**

The online version contains supplementary material available at 10.1186/s12877-026-06990-1.

## Background

Approximately 40% of older adults (aged ≥ 65 years) visiting European emergency departments (EDs) have frailty [1], which has become an increasingly prominent focus in emergency care research. Frailty is typically defined as a decline across multiple physiological systems accompanied by increased vulnerability to external stressors, which elevates the risk of delirium, falls, dependency, institutionalisation, and mortality [2]. Because patients living with frailty are at particular risk, geriatric emergency care guidelines recommend identifying frailty during ED visits to prevent adverse events and improve patient outcomes [3].

However, the extent to which frailty assessment is implemented in European emergency departments is unknown. Available data indicate variation: for example, the UK has widely implemented the Clinical Frailty Scale (CFS) [4], whereas some Swedish EDs use various instruments to assess frailty [5]. While many frailty assessment tools exist and aspects such as their prognostic value and reliability have been studied in emergency care [6], patients’ experiences of undergoing these assessments have received limited attention. Given that patients in EDs value holistic and person-centred care [7], understanding these experiences is particularly important as the assessment itself evaluates patients’ functioning, whereas being labelled as “frail” may be perceived negatively by some patients [8].

To our knowledge, only one study has specifically explored patients´view on frailty assessment in the ED. Blomaard et al. [9] investigated the Acutely Presenting Older Patient screening tool and found that although patients often had difficulty recalling the assessment, they generally regarded it positively. Patients believed the assessment contributed to holistic care and enabled early detection of geriatric problems.

Among the available tools is the Clinical Frailty Scale (CFS), which is one of the most widely used [10, 11] and is frequently evaluated in the ED [12]. As the CFS has been adopted as the regional frailty screening tool in our county (Region Östergötland), this study forms part of a broader evaluation of its practical use, while its widespread use also increases the relevance of the findings. The CFS is a nine-point scale validated for people aged ≥ 65 years and consists of clinical descriptions of a person’s level of functioning in daily life and cognitive status [13]. To determine the CFS score, healthcare staff must ask patients about their physical activity and level of functioning in instrumental and personal activities of daily living, such as housekeeping, shopping, banking, medication management, dressing, and personal hygiene. These questions are often not directly linked to the patient’s acute illness but relate to aspects that may be tied to integrity, requiring that the person pay attention to aspects such as one’s abilities and everyday life.

Despite its widespread use, no previous studies have been found that investigate patients’ experiences of being assessed with the CFS during an ED visit. Gaining a better understanding of these experiences may support ED staff by providing insight into how patients recognise a person-centred approach during frailty assessment, ultimately improving assessment practices and patient care.

### Aim

This study aimed to investigate ED patients’ experiences of frailty assessment using the CFS, including the factors shaping the experience.

## Methods

### Study design

This study adopted an exploratory qualitative descriptive design [14] inspired by the phenomenological perspectives of lived experiences (herein, patients’ experiences of frailty assessment in the ED context). Data collection included video-recorded clinical encounters in which CFS assessment was performed by regular ED staff, as well as subsequent semi-structured interviews with patients. The study was registered at ClinicalTrials.gov (identifier: NCT06621290) on September 30, 2024.

### Setting

Data were collected in the ED of a Swedish university hospital with approximately 50,000 visitors annually, approximately 40% of whom were adults aged ≥ 65 years. CFS assessment was performed in any single-patient room in the ED by a physician, registered nurse, or assistant nurse in charge of patients. CFS assessment has been part of the clinical routine in the ED since 2021 (i.e., three years prior to this study), and all employees have been instructed to adhere to this standard (i.e., to perform CFS assessment on patients aged ≥ 65 years) and have been encouraged to participate in an e-learning course on the use of CFS. More recently, employees have been educated about CFS assessment as part of their introduction to the ED. At the time of the study, there was no dedicated care pathway for older adults or any other specific measures for presumably frail patients; nevertheless, the CFS was part of the daily procedures in the ED.

### Criteria

Between 30 September and 5 November 2024, the two researchers in charge of data collection (EH and DE) addressed patients aged ≥ 65 years visiting the ED during daytime. To ensure perspectives from both robust and frail patients, and from individuals presenting with different types and severities of complaints, a purposive sampling strategy was used [14], aiming for variations in age, sex, frailty status, chief complaint, triage category, visiting hours, and staff workload. Patients who lacked the cognitive capacity to provide informed consent, had a critical condition (triage category 1), or did not speak Swedish (for whom an authorised interpreter could not be arranged) were excluded from the study. ED staff were included if they performed CFS assessments on patients deemed eligible for the study but were excluded if they were unwilling to participate.

### Data collection

Only when a patient meeting the study criteria was identified by the researchers was the staff in charge invited; if any member of the patient’s team consented, the patient was informed about the study, and written informed consent procedures were performed for staff and patients. At this point, staff were asked to indicate their perceived workload during the past hour on a scale of 1–6. If the patient was not already in a single room, they were relocated to another room; this was not advertised in the study information provided to the patient. A video recording device was set up at the head end of the patient’s trolley or bed (out of the way for any procedure), and the staff member then performed the CFS assessment, which was recorded. The recording captured both the patient and staff but did not focus directly on their faces. The next of kin was also present during the assessment. The CFS assessment was performed at any time during the patient’s ED visit, and the researcher started the recording when notified by the staff that the CFS was to take place and ended once it had been completed. Thus, no researcher was present when the CFS was performed. Following the CFS assessment, a semi-structured interview was conducted with each patient by a researcher; next of kin attending the CFS were welcome to be present during the interview but were not addressed by any questions. No staff members were present during the interviews.

To ensure consistency, the first two interviews were conducted jointly by two researchers (EH and DE), whereas all subsequent interviews were conducted individually by either of them. The researchers were dressed similarly to regular staff but introduced themselves as researchers and wore tags to clarify their role. An interview guide (Appendix 1) containing both primary questions and follow-up prompts was used. On average, the interviews lasted for 13 min (range: 5–24 min). Data analysis was commenced while collecting data, and participant recruitment continued until no new aspects of the emerging themes were identified [15]. All interviews were audio recorded using a digital voice recorder and transcribed verbatim using NoScribe software (NoScribe version 0.5, Kai Dröge, 2023), with final edits to ensure correct transmission by EH. All transcripts were coded for confidentiality and uploaded to DelveTool qualitative analysis software [16].

### Data analysis

The interview analysis followed the thematic approach by Naeem et al. [17]. Two researchers (EH and DE) conducted the analysis with regular involvement from a senior qualitative researcher (ACE).

To ensure a comprehensive understanding of the data, the transcripts were independently reviewed, and an initial naive understanding was documented. Subsequently, the transcripts were revisited to mark meaning units and identify keywords to capture recurring patterns or significant experiences. Related keywords were grouped into codes, resulting in a preliminary coding scheme. This scheme was independently applied to all transcripts, and discrepancies were resolved by revisiting the transcripts and audio files to ensure an accurate interpretation. The codes were then formed into themes that were refined and developed to ensure that they captured the essence of the data while remaining mutually exclusive and clearly delineated. The codes and themes were organised into a figure illustrating the patients’ narratives of how they experienced the CFS assessment and the factors that shaped that experience. This figure then served as a coding scheme when subsequently analysing video data.

The videos were first viewed individually and then collectively to gain a naïve understanding and to identify key events in the interactions between staff and patients. Codes generated inductively from the interview analysis were subsequently used as a lens for a deductive analysis [17] of the video data, in which observed events were coded in relation to patients’ described experiences. During this process, the coding scheme was iteratively refined, prompting a return to the interview data for deeper analysis. An example of how participant quotes, video observations, keywords, codes, and themes were linked is presented in Table 1.

**Table 1:** Examples from the data analysis process

Participant quotes	Observations from videos	Keywords	Subcodes	Codes	Themes
“You can tell that she is listening. She listens, and when I say something, she asks a little more about it.”	Staff use open-ended questions with follow-up prompts.	Being interested	Being listened to actively	Being involved in relationship- oriented communication	Shaping the CFS experience
“If I say it’s about being seen… almost.”	Staff create a supportive environment, saying things like, ‘It’s okay to cry.’	Validating the patient	Being invited to build rapport	Being involved in relationship- oriented communication	Shaping the CFS experience
“My visits to the hospital have always involved honest people… and then perhaps they are not. I have got the impression that the hospital is a good institution.”	Patients exhibited trust by engaging with staff questions and openly sharing aspects of their personal lives.	Trust	-	Trusting healthcare	Shaping the CFS experience
“I still consider myself vital. I’m not quite ready to admit that I struggle with things. Perhaps I’m a bit vulnerable.”	Descriptions of activities that are becoming more difficult, indicating functional decline.	Admitting	-	Acknowledging change	Reflecting on ageing

The final themes were synthesised collaboratively within the research group through an iterative, consensus-driven process, ensuring that all findings were integrated to provide a comprehensive understanding of patients’ experiences.

### Rigour and trustworthiness

Throughout the analysis process, the following strategies were employed [18]:

*Credibility* was assessed through a reflexive approach, in which the researchers pondered and recognised their preconceptions to avoid bias. Thus, the analytical steps were conducted separately before the joint discussion. The use of the two different data sources provides complementary perspectives and contributes to a comprehensive understanding of the findings.

*Transferability* was supported through purposive sampling, descriptions of participant attributes, and descriptions of the study settings.

*Dependability* was supported by maintaining an audit trail.

*Confirmability* was ensured through repeated revisits to the source data, regular analytical debriefings with a senior researcher, and peer debriefings.

### Research team and public involvement

The research team consisted of four researchers from diverse backgrounds in the nursing and medical sciences. The team comprised two male and two female researchers. Two senior researchers held PhDs in nursing and medical science, while the other members held master’s degrees in nursing science, one of whom was a doctoral student.

In this study, a public research partner who had completed a shorter research course at a local university was engaged. The research partner provided input throughout the study, contributing to the development of patient information materials, testing technology, and interview guides and provided an intuitive analysis of the transcripts as well as reflections on the study’s conceptualisation. The research partner was representative of the study’s aim, since of the same age group as the intended participants and had experience as a relative to an older person visiting the ED.

### Ethical considerations

The Swedish Ethical Review Authority approved this study (permit no. 2024-03729-01). The study was conducted in accordance with the Declaration of Helsinki [19]. Written informed consent was obtained from each participant (patient and healthcare professional) prior to the assessments and interviews. Before obtaining consent, we ensured that the study conditions and purpose were clarified, allowing extended time for processing the information and providing hearing or visual aids if necessary. All study data will be retained for at least 10 years and stored in accordance with applicable legislation. Digital data are stored in an encrypted and access-controlled storage space within Region Östergötland, and papers are kept in a locked space with no access for unauthorised persons.

## Findings

Twenty-nine patients were invited to participate in the study: however, eight declined owing to a lack of strength or interest in research studies, resulting in a final sample of 21 patients with varying ages, triage priorities, and frailty levels (Table 2). Some eligible patients were not invited because of staff declining participation. The primary reason for staff decline was reluctance to be video recorded while performing the CFS assessment, often because of concerns about not performing well. Overall, CFS assessment was performed by 17 ED staff members of different professions, ages, and work experiences (Table 3), with four staff members conducting two assessments each. CFS assessment lasted from 1 to 9 min, with a mean duration of 5 min. Appendix 2 provides a description of how the assessments were expressed in practice.

**Table 2:** Characteristics of the participating older adults: age, sex, frailty level (CFS), chief complaint, and staff’s estimated workload in the hour before the frailty assessment. CFS, clinical frailty scale

Participant	Age group	Sex	CFS (1–9)	Triage priority (1–5)	Chief complaint	Workload (1–6)
1	75–79	Male	3	3	Chest pain	3
2	90+	Male	5	3	Dyspnoea	2
3	65–69	Male	4	3	Extremity pain	1
4	80–84	Female	4	3	Dizziness	3
5	80–84	Female	2	3	Fever	2
6	65–69	Male	4	2	Abdominal pain	3
7	80–84	Male	5	3	Diarrhoea	3
8	70–74	Male	2	2	Neurology	1
9	85–89	Male	4	3	Haematuria	4
10	65–69	Female	1	3	Fever	1
11	75–79	Female	6	2	Fever	3
12	65–69	Female	4	3	Dyspnoea	2
13	80–84	Male	5	2	Head injury	2
14	80–84	Male	6	4	Extremity injury	3
15	75–79	Female	2	3	Syncope	2
16	70–74	Female	3	3	Dyspnoea	2
17	90+	Female	6	4	Dyspnoea	2
18	70–74	Female	3	2	Fever	4
19	80–84	Female	1	4	Extremity swelling	4
20	65–69	Male	5	2	Headache	4
21	65–69	Female	4	3	Abdominal pain	2

**Table 3:** Characteristics of the participating ED staff

	Staff (*N* = 17)
Age in years, mean (min–max)	38 (24–52)
Female, *n* (%)	13 (76%)
Male, *n* (%)	4 (24%)
Work experience in years, mean (min–max)	12 (1–22)
Registered nurses, *n* (%)	8 (47)
Assistant nurses, *n* (%)	5 (29)
Physicians, *n* (%)	4 (24)

Data collection and analysis were conducted in parallel. Recruitment continued until no new themes or subthemes emerged in the ongoing analysis, at which point thematic saturation was deemed to have been reached [15]. The dataset was considered sufficiently rich to capture variations in patients’ experiences.

**Fig. 1:**
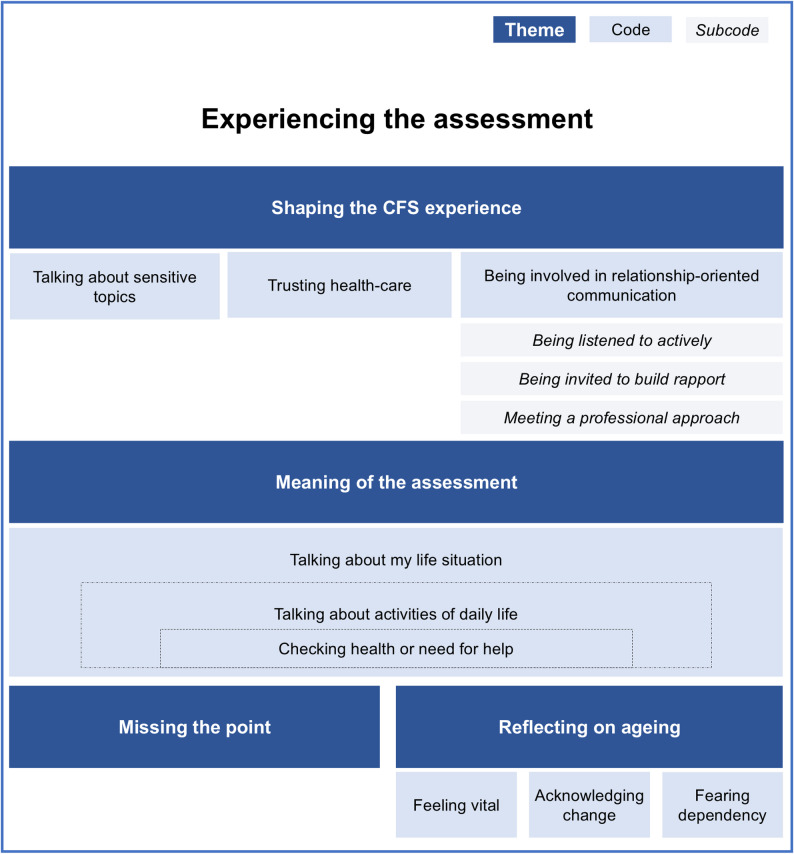
Overview of themes, codes, and subcodes illustrating how patients experienced the CFS assessment. Sensitive topics, trust in healthcare, and relationship-oriented communication were central factors shaping these experiences. Patients perceived the assessment’s meaning on different levels, relating to experiences of missing its point and to reflections on ageing

Participants described their general experiences with the CFS assessment in various ways. Positive experiences were marked by feelings of being cared for, finding the assessment relevant to important areas, and perceiving questions as easy to answer. Indifferent reactions included viewing the questions as reasonable or non-intrusive and simply appreciating a brief conversation. However, a few participants found the assessment strange, describing it as lacking a holistic perspective or including questions that felt illogical in the context of an acute care situation. These general experiences serve as a starting point for a deeper understanding of how patients experience CFS assessment in the ED. This understanding, drawn from both interviews and videos, is presented below in four themes - namely, *Shaping the CFS experience*, *Meaning of the assessment*, *Missing the point*, and *Reflecting on ageing* - each with corresponding codes and subcodes (Fig. 1 and in italics below), offering distinct nuances of the assessment process.

### Shaping the CFS experience

Participants framed several factors that influenced their experiences, one of which was their *sensitivity to certain topics* covered in the assessment. Although patients generally found the questions unproblematic to answer, they suggested that the questions addressed topics that could hit a nerve and acknowledged that discussing a challenging home situation might expose oneself. As one participant expressed, *“…they absolutely don’t want to… no*,* I’m not interested in that. I don’t want to talk about it. That’s none of their business” (p5).*

Another factor was the fundamental *trust in healthcare*, which influenced the willingness to share personal information. Participants highlighted both their general trust in the service and the importance of perceiving that the information shared during the assessments served a genuine purpose. They emphasised the confidentiality of healthcare: “*Of course … a nurse or a physician*,* I fully trust that it’s confidential. That’s why I dare to share. I don’t need to keep anything secret; it just is what it is” (p9)*. During the frailty assessments, patients trusted healthcare staff with personal information, informing the CFS assessment.

Participants identified the staff’s ability to build relationships and communicate as a key factor influencing their overall experience. They described being trustful and at ease when *being involved through relationship-oriented communication*. This was framed as a result of three subcodes: *Being listened to actively*, *Meeting a professional approach*, and *Being invited to build rapport*.

Patients experienced *being listened to actively* when staff showed interest, being calm and allowing the patient time to speak. It was seen in the videos as asking exploratory questions, repeating and summarising what the patient had said, and using nonverbal cues.

Patients also highlighted aspects of the staff *having a professional approach* which they suggested were reflected through clear questioning and the ability to guide the conversation. The factors associated with being professional were further evident in the videos in which the staff initiated the conversation by framing the upcoming assessment, showing knowledge of the patient and geriatric topics, and asked structured questions.

The third subcode, *being invited to build rapport*, encompasses interactions characterised by a sense of mutuality and connection in a relaxed tone and atmosphere, which encouraged the patients to open up and share their experiences. The video recordings revealed that the staff established rapport by introductions, humour, personal touch, validation of patients’ emotional states, and fostering mutual dialogue.

### Meaning of the assessment

Participants generally described the assessment as *talking about my life situation*, particularly in relation to their housing conditions and family circumstances. Video analysis confirmed that these two topics were the predominant starting points in most CFS assessments. Patients often described the assessment in relation to whether they lived alone or in a partnership. A participant living alone reflected “*We talked mainly about me… people who are alone might be more vulnerable to things…they might be more easily affected mentally and physically than when living in a family” (p8).* In contrast, patients who lived with their partners often described the assessment in terms of them as a couple: *“She asked whether we could manage on our own or if we needed help at home” (p12).* This was also recurrently shown in the videos, indicating that the frailty assessment of individual patients was sometimes influenced by their perception of what they and their partners were capable of.

Specifically, the assessment was perceived as *talking about activities in daily life*, focusing on the ability to perform such activities and the level of physical activity. This was reflected in videos with conversations about mobility, cleaning, personal hygiene, dressing, cooking, shopping, transportation, medication management, finances, and exercise.

When the participants reflected on the underlying aspects of the assessment, they generally suggested that its purpose was to identify any need for assistance, which was compiled in the code *checking health or need for help*. Some patients viewed it as an evaluation of basic health, whereas others were confused about the overall purpose of the assessment. In the videos, staff members introduced the assessment in various ways before initiating it; it either was described as an assessment, evaluation, or measurement or was more generally framed as *“asking a few questions about how you manage your daily life”* or “*to see if there’s anything else we need to help you with”*.

### Missing the point

Based on the varying understandings of the assessment described above, this theme captures how participants described uncertainty about whether the assessment led to further action, or what its conclusion was. Video analysis suggested that only one patient was informed about the expected outcomes of the assessment and the subsequent involvement of other team members or healthcare units, whereas in most cases, no message regarding further actions was provided. This lack of clarity was echoed by participants who reported uncertainty about the consequences of the assessment, including those who felt they understood its purpose. As one participant expressed, “*I miss a clear direction for what comes next. Of course*,* it’s important to know whether I live alone or in an apartment or house*,* whether I have a home alarm or not… and so on. I understand all that. These are the basics*,* but I thought we were supposed to move forward” (p11)*.

### Reflecting on ageing

One aspect of patients’ experiences with frailty assessment was that it initiated different reflections on the ageing process, which were captured in three codes: *Feeling vital*, *Acknowledging change*, and *Fearing dependency*. In both the interviews and the observations, patients often expressed feeling vital, due to being active and/or managing daily life. During the assessments, staff sometimes acknowledged these aspects (“you are very energetic”), and patients responded by reinforcing this self-image. This interaction appeared to affirm the patients’ positioning of themselves as vital and “not frail”, which was also evident when they reviewed the CFS levels and located themselves at the “non-frail” end of the scale (irrespective of whether they were assessed as living with frailty).

Several participants were *acknowledging change* when reflecting on the loss of certain abilities during both the assessment and interview. This included a desire to talk about their former life, and what they used to do in the past, as well as observations of change, with a sort of recognition to themselves: “*Maybe you … realise that you may not be at the top anymore” (p15).*

During both assessment and interview, the participants reflected on *fearing dependency*. They expressed a desire to remain as independent as possible for as long as possible, along with anxiety about having to accept help: *“That they (healthcare and social care staff) take away… your ability to do things too quickly. You need more and more help*,* it’s not that… but you want to wait as long as you can do things. Ordinary things. Things you need in your life. You should have the chance to do them. It shouldn’t be taken from you too soon. That’s how it is.” (p13)*. The reflections evoked concerns about practical issues such as the availability of care services for older people in the future, or whether their financial situation would preclude access to such services. For some, thinking about dependency also triggered worry and fears about losing autonomy and dignity, and even considerations about life itself if increased dependence became unavoidable.

## Discussion

This study explored older ED patients’ experiences with frailty screening using the CFS. Most participants reported positive or indifferent experiences, aligning with findings from Blomaard et al. [9], who found exclusively positive patient attitudes towards the “Acutely Presenting Older Patient” screener. However, our findings add that older ED-patients can view the assessment as strange or illogical. Overall, we suggest that modifiable factors such as clearly explaining the purpose and consequences of the assessment and using relationship-oriented communication may provide a more person-centred perspective in frailty assessment in the ED and prevent negative experiences.

In our study, CFS assessments took 1–9 min (mean 5), which is longer than previously reported durations of half to one minute [20, 21] but likely reflects real-world practice where patient complexity and communication needs vary. Although brief, our findings suggest that rapport can still be established when staff use relationship-oriented communication. We found that communication skills were central to the experience of frailty assessment, enabling ED staff to create positive encounters and overcome barriers such as sensitive topics or preexisting mistrust in the healthcare system.

The observations of active listening in our study resonate with the findings of Hermann et al. [22], who also describe rapport as an outcome of a sense of chemistry or connection - something staff in this study established through showing interest in the patient as a person and adding a personal touch in the conversation. Further, we found that staff demonstrated professional expertise by being knowledgeable about the patient or geriatric issues - an aspect previously identified as trust-building in a study by Pavedahl et al. [23].

Trust in healthcare staff is associated with patient satisfaction, health-promoting behaviours, and symptom severity [24] and forms the foundation for relationship-oriented communication [23, 25]. In turn, patient-centred communication is essential for cultivating trust [25]. This aligns with patients’ descriptions in the present study, where trust in healthcare influenced their willingness to be open during assessments. Since both our results and those of others [26], indicate that relationship-oriented communication promotes patients’ trust and openness, this has practical implications for ED practice, as it may facilitate more accurate frailty assessments. However, recent qualitative research on nurses’ relational work in EDs suggests that relational care is often optional and rarely discussed [27]. Supporting and making relational work more visible - for example, through training and reflection - may promote trust-building communication in practice.

Patients living with a partner frequently adopted a collective “we” perspective during assessments, challenging ED staff’s focus on an individual evaluation. This dynamic may be related to staff framing assessments in home contexts, prompting shared responses. This points to a discrepancy between how the CFS is viewed by healthcare professionals and how the assessment may be perceived by patients. While staff use the CFS as a risk stratification tool, patients may understand the questions as an exploration of needs within their everyday context, sometimes interpreted as an offer of help. While contextual insights remain valuable as they provide a holistic picture of the individual, clearer communication about the purpose of the assessment could prevent ambiguity.

The purpose of an assessment is closely linked to the actions it leads to for the patient. Previous studies have shown that both older adults [28] and care providers [29, 30] view frailty screening as meaningful only when it results in tangible action. For example, Blomaard et al. [9] found that some patients reported improved care because of APOP screening, a structured approach that includes both assessment and targeted follow-up actions. However, our study demonstrated a communication gap between patients and staff regarding the outcomes of frailty assessments (i.e. what actions will or will not follow). This communication deficiency may stem from ED staff occasionally lacking a comprehensive understanding of the specific actions that should follow such assessments [31], which can result in patients missing the point. This lack of clarity may reflect the absence of guidelines or clinical routines at the ED where the study was conducted; however, certain measures (e.g. targeted nursing care, early discharge planning, medication adjustment) may nevertheless have been undertaken without the link between the frailty assessment and these measures being made explicit to the patient. This highlights the potential value of ensuring mutual understanding and clarifying the relevance of the assessment of care decisions by summarising key points and discussing potential actions with the patient. In cases where no immediate action is needed (such as when a patient is robust), our findings suggest that clearly communicating this can be equally important for transparency and clarity. When further discussion with colleagues or other healthcare units is required, informing patients and reassuring them about follow-up can help maintain trust and engagement in their care [7].

Based on the frailty assessment, the study participants reflected on different dimensions of ageing. A scoping review of older adults’ views on successful ageing [32] highlighted the importance of maintaining a sense of vitality by focusing on independence and abilities that remain intact, at least to some extent. Such approaches to ageing were also evident among patients in our study, reflecting a salutogenic perspective that may sometimes be overlooked by healthcare professionals during frailty assessment. Further, previous research shows that patients often perceive the concept of frailty as negative [8], perhaps related to how it contrasts with aspects older adults associate with successful aging [32]. How frailty can be discussed with older patients without provoking stigma or unnecessary concerns remains an important issue for clinical practice.

### Limitations

This study was conducted in a single ED, in a Swedish context, and in daytime hours only. Although another first language than Swedish was represented in the sample, no participants required an interpreter. Patients with cognitive impairment were excluded for ethical reasons; however, these patients constitute a significant proportion [33] of the potential participants. A range of chief complaints was included, but because data collection coincided with a period of high prevalence of infections, these complaints came to dominate slightly. Although both robust individuals and patients living with frailty were included, those who were most severely frail could not be recruited. In this setting, such patients present less frequently, and when they did, they were excluded based on predefined criteria. Taken together, these factors may influence the transferability of the findings to other settings.

In addition, several staff members chose not to participate, which may have introduced selection bias. Eight eligible patients also declined participation due to fatigue or lack of interest. Although they did not differ from participants in any objective way, they may have been more unwell or less willing to engage in conversation, meaning that some perspectives may be underrepresented.

Finally, the involvement of interviewing researchers who worked within the ED and were engaged in implementing the CFS may have influenced the results by introducing observer bias, for example through expectations or familiarity with the setting, although steps such as reflexivity and researcher triangulation were taken to minimise this.

## Conclusion

Most older patients in the emergency department reported positive or indifferent experiences of the CFS assessment, underlining the importance of relationship-oriented communication for building trust and ensuring quality frailty assessment. However, patients did not always understand the purpose of the assessment or what actions followed, highlighting the need for clear explanations of both its purpose and potential outcomes.

## Supplementary Information

Supplementary Material 1.

Supplementary Material 2.

## Data Availability

The data supporting the findings of this study consist of qualitative interviews and video recordings which contain personal information. Due to privacy and ethical concerns, these data cannot be made publicly available. However, anonymised excerpts from the interviews may be available from the corresponding author upon reasonable request.

## Abbreviations

ED: Emergency Department
CFS: Clinical Frailty Scale
