# Supplementary material for: Being screened for frailty in the emergency department: the voice of patients in an exploratory qualitative study
Source: BMC Geriatr. 2026 Jan 16;26:144. doi: 10.1186/s12877-026-06990-1 (PMC12862903; doi:10.1186/s12877-026-06990-1)
Supplement: Supplementary file 1 — Supplementary Material 1. [file 12877_2026_6990_MOESM1_ESM.docx]

| **The semi-structured interview guide** | |
| --- | --- |
| **Primary questions** | **Examples of probing questions** |
| Could you please tell me about the conversation with the nurse/assistant nurse/doctor that you just had?  *If subject has difficulties remembering – hook:* What did you perceive it was about? | - What happened in the conversation? - Is there any particular part that you remember? - What do you think makes it stand out? - Is there any other part that you also remember? (can be asked multiple times) |
| If the person doesn’t mention all parts: I’d like to hear a bit more about the conversation. A conversation like this can include questions about… (activity, shopping, medications, housework, showering, getting dressed) | - Is that something you talked about? - What do you think about it? |
| How did you experience the conversation? | - Would you have wished for anything in the conversation to be different? - What do you take away from the conversation? |
| *When you arrive at the emergency department, many different assessments are made. In this case, the assessment aims to evaluate your general health condition and whether it poses risks to your recovery that staff need to plan measures for. We call it frailty or non-frailty. Show the scale and explain it.* | |
| What are your thoughts about the scale | - When you now think back on the conversation, what do you think about? - Would you like to tell me more about your thoughts? |
| *If the patient wants to know what score he/she received and what measures might be relevant, explain that you don’t have knowledge of that. Ask if he/she would like to know, in which case you can request the responsible staff to follow up on it* | |
