# Supplementary material for: Being screened for frailty in the emergency department: the voice of patients in an exploratory qualitative study
Source: BMC Geriatr. 2026 Jan 16;26:144. doi: 10.1186/s12877-026-06990-1 (PMC12862903; doi:10.1186/s12877-026-06990-1)
Supplement: Supplementary file 2 — Supplementary Material 2. [file 12877_2026_6990_MOESM2_ESM.docx]

| **Aspects** | **Observations** | **Variations** |
| --- | --- | --- |
| Introduction | Staff commonly introduced themselves and confirmed the reason for the ED visit. Permission was in several cases sought to perform an assessment or to “just ask a few questions". | The assessment was conducted at different points during the ED visit: some at the initial encounter, most after approximately one hour, and two more than ten hours after arrival. If the staff member and patient had met previously, no formal introduction was made. |
| Framing the assessment | When introducing the assessment to patients, staff used different formulations, including asking how the patient was managing at home, inquiring about the home situation to check for support needs or for later comparison, explaining that the assessment involved evaluating frailty using a scale, and presenting frailty level as a factor to be considered in care decisions when higher scores were obtained. | Most staff members sat down next to the patient, while a few remained standing. Body language such as maintaining eye contact, smiling, and leaning forward was used. |
| Targeted questions and conversations | The assessment included questions related to living arrangements and social situation, physical activity. All videos included questions whether they had any assistance to perform activities of daily living Questions were typically phrased in everyday language and focused on the patient’s habitual functioning, using concrete examples (e.g. managing personal care, shopping, household tasks, medication management, mobility, and use of home care services or assistive devices). Staff conveyed professionalism through structured questioning and knowledge of geriatric issues. Rapport was fostered by introducing a personal touch into CFS conversations and by using humour. | The number of targeted assessment questions varied between eight and approximately twice that, depending on the patient’s level of functioning. Patients perceived as very vital were identified early and required fewer questions, whereas patients with lower levels of functioning prompted a broader set of follow-up questions. Conversations ranged from being limited to assessment questions to including patients’ life stories or validating their emotional state through words and body language. |
| Conclusion of the assessment | The assessment often ended with a brief summary of what had been discussed (e.g. ‘I hear that you …’). Some staff also concluded with a plan, which in most cases referred to the ongoing emergency visit rather than the assessment itself. In some meetings, information was provided about where to seek help if needed. | Endings varied from brief confirmations (e.g. ‘I have a good picture now’) to slightly more extended summaries. |

**Appendix 2.** Summarises observed features of how the frailty assessment was conducted during the ED encounters, based on video-recorded observations.
